# Complete Genome Sequence of a Chinese Highly Pathogenic Porcine Reproductive and Respiratory Syndrome Virus That Has a Further Deletion in the Nsp2 Gene

**DOI:** 10.1128/genomeA.01770-15

**Published:** 2016-02-18

**Authors:** Guobiao Ji, Yingying Li, Feifei Tan, Jinshan Zhuang, Xiangdong Li, Kegong Tian

**Affiliations:** a College of Animal Science and Veterinary Medicine, Henan Agricultural University, Zhengzhou, People’s Republic of China; b National Research Center for Veterinary Medicine, High-Tech District, Luoyang, People’s Republic of China; c Veterinary Diagnosis Center and OIE Porcine Reproductive and Respiratory Syndrome Laboratory, China Animal Disease Control Center, Chaoyang District, Beijing, People’s Republic of China

## Abstract

Here, we report the complete genome of a Chinese highly pathogenic porcine reproductive and respiratory syndrome virus (HP-PRRSV) characterized by a further 29-amino acid (87 nucleotides) deletion in its Nsp2-coding region compared to the prototype of the HP-PRRSV JXA1 strain.

## GENOME ANNOUNCEMENT

The highly pathogenic porcine reproductive and respiratory syndrome virus (HP-PRRSV) was classified into genotype 2 (North American genotype) of PRRSV which belongs to the order *Nidovirales*, family *Arteriviridae*. Since the first report of HP-PRRSV in 2006, it has become the predominant circulating field strain in Chinese swine herds (1). Compared with other classical type 2 PRRS viruses that emerged before 2006, the HP-PRRSV represented by JXA1 has a discontinuous 30-amino acid (aa) deletion in the Nsp2 gene (amino acids 481 and 533 to 561) (2). Since the Nsp2 gene is the one of most variable regions among PRRSV isolates, more mutations including deletions were reported spanning the whole gene (3, 4). In this study, we announce the genome of a novel HP-PRRSV strain GZgy15-1 that has a further 29-amino acids (amino acids 488 to 516) deletion in its Nsp2 gene compared with JXA1, which was not previously reported.

The GZgy15-1 strain was isolated from the hilar lymph node sample of a piglet that showed clinical respiratory syndromes in the Guizhou Province, west China. Eighteen pairs of primers were used to amplify overlapped fragments to map the virus genome (5). The PCR products were purified and cloned into pGEM-T Easy Vector (Promega) and were sequenced with an automated sequencer (Genetic Analyzer 3730XL, Applied Biosystems). The complete genomic sequence of GZgy15-1 is 15,271 nucleotides (nt) long, with a 5′-untranslated region (UTR) of 189 nt, followed by a poly-protein precursor coding sequence and a 3′-UTR of 188 nt. Open reading frame 1a (ORF1a) and ORF1b of virus are 7,335 and 4,383 nt in length, and ORF2 to ORF7 are 771, 765, 537, 603, 525, and 372 nt in length, respectively.

The comparison of GZgy15-1 with JXA1 by the whole-genome BLAST technique shows that there is a further deletion of 87 nt (1,464 to 1,546 nt) in the Nsp2 region. The complete genomic sequence alignment of GZgy15-1 shared 97.1%, 88.4%, and 60.9% nucleotide identities with the HP-PRRSV prototype JXA1, the American prototype VR-2332, and the European prototype Lelystad virus (GenBank access numbers EF112445, U87392, and M96262), respectively.

The new-emerging GZgy15-1 sequence data updates the database of HP-PRRSV genome sequences and could aid in the study of the genetic diversity and evolutionary characteristics of HP-PRRSV in China.

### Nucleotide sequence accession number.

The complete genome sequence of the GZgy15-1 strain is available in GenBank under the accession number KT358728.

## References

[1] Feng Y, Zhao T, Nguyen T, Inui K, Ma Y, Nguyen TH, Nguyen VC, Liu D, Bui QA, To LT, Wang C, Tian K, Gao GF 2008 Porcine respiratory and reproductive syndrome virus variants, Vietnam and China, 2007. Emerg Infect Dis 14:1774–1776. doi:10.3201/eid1411.071676. 18976568 PMC2630730

[2] Tian K, Yu X, Zhao T, Feng Y, Cao Z, Wang C, Hu Y, Chen X, Hu D, Tian X, Liu D, Zhang S, Deng X, Ding Y, Yang L, Zhang Y, Xiao H, Qiao M, Wang B, Hou L, Wang X, Yang X, Kang L, Sun M, Jin P, Wang S, Kitamura Y, Yan J, Gao GF 2007 Emergence of fatal PRRSV variants: unparalleled outbreaks of atypical PRRS in China and molecular dissection of the unique Hallmark. PLoS One 2:e526. doi:10.1371/journal.pone.0000526. 17565379 PMC1885284

[3] Zhou Z, Li X, Liu Q, Hu D, Yue X, Ni J, Yu X, Zhai X, Galliher-Beckley A, Chen N, Shi J, Tian K 2012 Complete genome sequence of two novel Chinese virulent porcine reproductive and respiratory syndrome virus variants. J Virol 86:6373–6374. doi:10.1128/JVI.00663-12. 22570245 PMC3372213

[4] Zhou F, Zhao J, Chen L, Chang HT, Li YT, Liu HY, Wang CQ, Yang X 2015 Complete genome sequence of a novel porcine reproductive and respiratory syndrome virus that emerged in China. Genome Announc 3(4):e00702-15. doi:10.1128/genomeA.00702-15. 26159524 PMC4498110

[5] Zhou Z, Ni J, Cao Z, Han X, Xia Y, Zi Z, Ning K, Liu Q, Cai L, Qiu P, Deng X, Hu D, Zhang Q, Fan Y, Wu J, Wang L, Zhang M, Yu X, Zhai X, Tian K 2011 The epidemic status and genetic diversity of 14 highly pathogenic porcine reproductive and respiratory syndrome virus (HP-PRRSV) isolates from China in 2009. Vet Microbiol 150:257–269. doi:10.1016/j.vetmic.2011.02.013. 21411250

